# Supplementary material for: Assessment of the functional efficacy of root canal treatment with high-frequency waves in rats
Source: PLoS One. 2020 Sep 29;15(9):e0239660. doi: 10.1371/journal.pone.0239660 (PMC7523981; doi:10.1371/journal.pone.0239660)
Supplement: Attachment — Submitted filename: review.docx [file pone.0239660.s001.docx]

The aim of this paper is interesting and the study may provide a new non-surgical treatment for apical periodontitis, however some flaws need to be addressed.

**Abstract:**

1. Line 38-40, the authors concluded that “high frequency wave irradiation promoted healing of the periapical lesion through increased expression of *VEGF*, *FGF2* and *TGF-β1*”, however, in the part of ”Immunohistochemical observation” (methods), the samples were stained with specific antibodies against *IL-1β*, *TGF-β1* and *FGF2*. Please explain how these results lead to the conclusion described above.

**Introduction:**

2. Line 48-49, “It has been reported that the average success rate for initial root canal treatment (RCT) was lower than that of pulpectomy”. This sentence is quite confusing. Please explain this in detail.

**Methods:**

3. In the parts of “Root canal treatment in rats”, line 128-129, “HFW irradiation was applied three times each to the periapical lesion and the inside of the root canal immediately before the root canal obturation”, however, in the part of “Quantification of bacteria in the root canal”, line 150-151, “HFW irradiation was applied three times to the periapical lesion and the root canal after the root canal preparation”. Are these two time point the same?

4. Line 153-154, “Immediately following the RCT, 10 rats were sacrificed and the mandibular first molars were extracted”, the rats were sacrificed and bacteria were collected immediately following the RCT, however, in the corresponding part of result, line 267, “At 4 weeks after RCT”. Authors described the ATP and PCR results for “Quantification of bacteria in the root canal” was from samples from 4 weeks after RCT. Please confirm the descriptions and make the unification.

**Results:**

5. Line 255-258, “Compared with the control group, the volume of the periapical lesions in the mesial root was significantly smaller in the irradiated group at 2 weeks after the RCT (p = 0.009), and in the non-irradiated group at 6 weeks after the RCT (p = 0.013, Fig. 2)”, authors described that the volume of periapical lesions was significantly smaller in non-irradiated group at 6 weeks after RCT, but form Fig. 2, we could find that at 2 weeks after RCT, the volume of periapical lesions was also significantly smaller in non-irradiated group when compared with the control group (“a” and “b” meant significant difference). Please confirm the result.

6. Line 333-335, “In rat osteoblasts, the expression of *FGF2* was significantly higher in HFW irradiated osteoblasts than in non-irradiated osteoblasts at 3 days (irradiation applied 5 and 10 times, p = 0.010) and 5 days (irradiation applied 10 times, p = 0.013)”, the authors obtained the result that the expression of *FGF2* was significantly higher in HFW irradiated osteoblasts than in non-irradiated osteoblasts at 5 days with 10 times irradiation, however, from Fig. 7(b), we could observed that both for irradiation with 5 times and 10 times at 5 days, expressions of FGF2 showed no significant difference (the same letter “a” meant no significant difference). Please confirm the result.

7. Line 344-345, “By comparing the expression level of (a) *FGF2*, (b) *VEGF*, and (c) *TGF-β1* in rat fibroblasts, and (d) *FGF2*, (e) *VEGF*, and (f) *TGF-β1* in rat osteoblasts”, the authors described that (a), (b) and (c) meant the expression level in rat fibroblasts, and (d), (e) and (f) meant the expression level in rat osteoblasts. However, in Fig. 7, we found t (a), (b) and (c) meant the expression level in rat osteoblasts, and (d), (e) and (f) meant the expression level in rat fibroblasts, which was quite contrary to the description. Please confirm it.

**Discussion:**

8. The irradiation times in all parts of *in vivo* were consistent (three times each to the periapical lesion and the inside of the root canal), however, in the in vitro assays, the irradiation times were various (15 times for biofilm discs, 5 and 10 times for cells). The authors should discuss why they chose these different irradiation times and whether the different irradiation times affected the therapeutic effect.
