# Supplementary material for: Assessment of the functional efficacy of root canal treatment with high-frequency waves in rats
Source: PLoS One. 2020 Sep 29;15(9):e0239660. doi: 10.1371/journal.pone.0239660 (PMC7523981; doi:10.1371/journal.pone.0239660)
Supplement: Attachment — Submitted filename: Response to Reviewers.docx [file pone.0239660.s002.docx]

Reviewer #1: The purpose of this study was to investigate the potential effects of high frequency wave (HFW) irradiation in healing outcomes of experimental apical periodontitis in vivo and in vitro conditions. Several methodological concerns and lack of clarity in details jeopardize proper interpretation of study findings. The results need careful interpretation in light of the lack of translatability of methods to be used in clinical practice, and the lack of a true biofilm in the experiments. It is not clear why the authors went into great lengths with the in vivo study to then turn into in vitro studies to assess expression of growth factors. It would make more sense to use periapical lesions/normal tissues from the animals for these assays.
Extensive grammatical and technical revisions are necessary.

High-frequency waves (HFW) are already applied clinically in medical field for fractures and other conditions, however, it is an indirect application from outside of the skin surface and not a direct application to the lesion. In the dental field, it is also used indirectly in cases of TMJ disorders and post-implant treatment. There have been no methods which utilize the direct application of HFW by inserting electrode to bone defects, and novelty of this direct application of HFW to the lesion is high. Moreover, HFW is applied as a complementary treatment for regenerative therapy but never applied in infectious disease as a complementary treatment, and this is the very first research to indicate biologically and scientifically that HFW can promote healing through stimulating the immune system. For all the diseases that develop in the host-parasite balance, this study showed the potential for clinical application of HFW direct irradiation and we do think it is of high significance.

This experiment was conducted in vivo, because replicating the healing model of HFW root canal therapy in rats was one of our targets. The original root canal treatment model of rats was the world’s first in vivo mouse model developed by our group, and it can reproduce human situation by performing RCT under a rubber dam isolated sterile condition. By creating a rat HFW root canal therapy model modified from original rat RCT model, we thought it can be investigated the mechanism of HFW therapy which is already reported the efficacy in promoting healing in human clinical studies but no mechanisms are indicated for now. Therefore, in this study, we created the rat HFW root canal therapy model first and then we verified the healing process and its mechanism of action.

As this reviewer pointed out, our in vitro experimental results caused various confusions so we clarified the point by focusing on in vivo experiments and modified the body substantially. We appreciate your comments.

line 48 – What are the authors trying to say with: “It has been reported that the average success rate for initial root canal treatment (RCT) was lower than that of pulpectomy [2–5].” Pulpectomy is part of RCT and these procedures are not alternatives of each other. This does not make sense.

This part made readers confused so we deleted.

Line 48- Revise ‘tight’ obturation with ‘dense, hermetically-sealed’ obturation

Following your suggestion, we changed the phrase to ‘dense, hermetically-sealed’ obturation.

Line 58 – a reference is needed at the end of the sentence

That part has been corrected and references have been added.

Line 59 – HFW is NOT an accepted method for NSRCT, therefore the authors should limit their purpose to analyse HFW as an ADJUVANT in NSRCT procedures.

As per your comment, HFW plays a complementary role in NSRCT procedures. So, we have added the word "adjuvant" to that part. We aimed to investigate the complementary effect of HFW irradiation in addition to NSRCT, which is mechanical / chemical removal of the infectious sources.

Line 113 – add some brief details of the lesion induction protocol for clarity. Were lesions induced on the right and left side of all animals in both irradiated and non irradiated groups? Clarify

Lesions were induced on the right and left side of all animals in both irradiated and non-irradiated groups. This part has been revised as “Periapical lesions were induced by exposing the pulp of all the right and left mandibular first molars.” to avoid confusion.

Lines 118-120 – Please revise this sentence. “enlarging’ a canal means the apical diameter established during instrumentation. I assume “enlarged to the level of 1.0” refers to working length distance to the apical foramen, as determined by an electronic apex locator (which the authors erroneously call ‘root canal meter’. The size of apical enlargement, which should be standardized between groups and of utmost importance to this study however is not mentioned in the text.

We revised that part as below.

“The working length was set to the level of 1.0 as indicated by an electronic apex locator (Root ZX, J. Morita Manufacturing) using K-files (Dentsply Maillefer, Ballaigues, Switzerland) and the root canal was then enlarged up to a #20 file.”

Justify the use of 0.5ml of 2.5% NaOCl
It is reported that 2.5% NaOCl has antimicrobial effects. We added the reference (30) there.

In relation to the amount of irrigant, average working length of a rat was 4mm and human molar was 12mm so volume ratio becomes 1:27. Also from the same reference, irrigation was performed with 5ml at each time, in total 20ml for human root canal. We irrigated with 0.5ml each time, from #8 K-file to #20. Calculated human data to rat, 0.19ml of irrigant is needed each time, and the amount used for rat irrigation was sufficient.

Justify the use of single cone obturation technique and provide details of the gutta percha cones used

Randomized controlled trial (31) reported that the obturation of the root canal using file-matched single cones presented a similar quality to that obtained with the lateral condensation technique. Similar healing rates of apical lesions were also observed so we added that reference.

Line 126, add number of rats divided in irradiated and non irradiated groups each

We added the number of rats for each experiment. N=6 each for micro-CT scanning and immunohistological observations, and N=5 each for bacterial quantification. Detailed information for each group is shown in the table below.

Line 128 – HFW was irradiated – was this through the foramen? How far out into the periapical lesion and how was this measured? Clarify

We irradiated HFW both outside of foramen and inside the root canal (total of three times). We irradiated once at 1mm over from the working length, and twice at -2mm from the working length since the average canal length of a rat is 4mm. This was consistent with human clinical conditions (26).

Line 203 – Biofilm formation was described to occur using 7-day polymicrobial cultures. However true biofilms have been shown to require a minimum of 21 days of culture. Hence the authors cannot state that a biofilm was used, only a polymicrobial mixture.
The results presented do not provide clear direction as if the images in Fig. 2 and 4 come from one animal or if representative sections of various animals are presented.

According to Costerton et al (1997), biofilm is defined as “Bacteria that attach to surfaces aggregate in a hydrated polymeric matrix of their own synthesis to form biofilms”, and there are no requirements for the culture period. The conditions for “true” biofilms, must be *in vivo/in situ* and our *in vitro* or any *in vitro* biofilm model cannot be “true” biofilms. However, *in vitro* biofilms are very important to understand the basic characteristics, and with the experimental limitation, we sometimes have to use an *in vitro* model.

This time we revised the whole manuscript to focus on the in vivo part so we deleted the in vitro biofilm part. As shown before, rats used for micro-CT analysis and immunohistological observation are different individuals. For better understanding, we added the micro-CT images in Fig. 2.

Line 351 – this does not reflect the study purpose stated in the introduction

We changed the study purpose in the introduction to “In the present study, we developed a new high-frequency therapy model in rats based on Yoneda et al. [9], with some modifications, to be used to elucidate the mechanism of action of HFW therapy in apical periodontitis. The influence of HFW as a supplement to conventional root canal therapy on periapical lesions was evaluated by three-dimensional micro-CT analysis, bacteriological evaluation and immunohistochemical analysis.” And the conclusion part was changed to “Our study focused on the essential conditions in situ / in vivo, and our findings indicate that HFW may be effective in stimulating immune-response cells in the model for treatment of infected root canals in rats. Of particular interest is the effect of HFW on host cells.” to reflect the study purpose.

The conclusions are not supported by the data presented – e.g. “it was proved that HFW irradiation promoted periapical lesion healing”. The authors fail to recognize that the success of endodontic treatment alone is >90%, therefore the HFW if any, might have helped the process by stimulating immune-response cells.

As we understand, only NSRCT can lead to healing. This time we found the healing speed to be significantly faster with HFW irradiation compared to NSRCT itself, so we think HFW irradiation is beneficial as an adjunctive therapy with NSRCT. We modified those parts throughout the manuscript for better understanding.

Lastly, HFW cannot be suggested as a new treatment for apical periodontitis as standalone. Root canal treatment is still necessary, as shown by their own results in which HFW had little to no activity on bacteria. Therefore the conclusions need to be toned down.

As per the previous comment, we understand HFW root canal therapy cannot be a standalone treatment for apical periodontitis. So this time we emphasize HFW is an “adjuvant” therapy used with NSRCT.

Figure 1 – add the number of animals used in each experiment

We added the number.

Figure 2 – include representative microCT images for each test and control site in irradiated and non irradiated groups that correlate with the results graph presented. Ideally the microCT images in which the obturation of the root canal can be visible as well as the periapical lesion size must be included.

We added representative micro-CT images for each group.

Figure 4 – include lower magnification images showing the level of root canal obturation in irradiated and non irradiated groups.

Unfortunately, we don’t have the lower magnification images showing the level of root canal obturation and the lowest magnification we could obtain was x40, we have images of the larger field of view (IL-1β and HE staining) so here we show them below. The level of root canal obturation could also be presumed from the micro-CT images added in Fig. 2.

Reviewer #2: I would like to thank the authors for submitting their article to PLOS ONE. It was my pleasure to review the manuscript entitled “Assessment of the functional efficacy of root canal treatment with high frequency waves in rats”. Undoubtedly, a lot of work was done to carry out this study and I commend the authors on their efforts in preparing the manuscript. Several analyses were performed in this study, mixing in vivo and in vitro assays, which makes it too long, difficult to read and sometimes even confusing. I suggest the authors to divide this paper in two: in vivo and in vitro assessment. Although the manuscript is very interesting, there are significant shortcomings that deter my enthusiasm.

As this reviewer pointed out, our previous manuscript was long and made readers confused about in vitro and in vivo data. This time we modified the whole manuscript concentrating on in vivo experimental part to clarify the point. We focused on investigating the mechanism of High-frequency wave (HFW) therapy which is already reported the efficacy in promoting healing in human clinical studies but no mechanisms are indicated for now by using rat animal model. So, in this study, we created the rat HFW root canal therapy model first and then verified the healing process and its mechanism of action.

We appreciate your comments.

- Overall, the entire manuscript needs to be revised for proper English/grammar/syntax/spelling

We modified the whole manuscript and English proofreading was performed by a native English proofreader.

Introduction
- "RCT often 50 fails because the anatomical complexity of the root canal system, which includes lateral branches and isthmuses". This is information is not correct.. the success rate of primary root canal treatment is high, around 90-95%

This part made readers confused so we deleted it.

- "This study focused on high frequency wave (HFW) apical therapy, which is a nonsurgical treatment for apical periodontitis" This kind of sentence should not be in this section

We deleted that part and modified it to show that HFW plays a complementary role to NSRCT procedures.

Methods
- This section is confusing and also the experiments need to be better described instead of using the expression "according to the method"

We added the explanation to try to describe the methodology more.

- Did the authors measured the periapical lesions before root canal treatment?

No, we didn’t measure the size of the lesions before we started RCT. We understand it is ideal to measure it before starting RCT and re-measure after RCT, however effective time for general anesthesia is around 30min and if we use the general anesthesia for consecutive days, rats’ fatality rate rises. Therefore, we had to compromise to set the baseline (0 week) at just after root canal obturation.

- Page 8, line 118. Eletronic apex locator instead of electrical root canal meter

We changed that part following your suggestion.

- Which file was used to enlarge the root canals?

We used K-files from Dentsply. We modified that part to make it clear as below.

“The working length was set to the level of 1.0 as indicated by an electronic apex locator (Root ZX, J. Morita Manufacturing) using K-files (Dentsply Maillefer, Ballaigues, Switzerland) and the root canal was then enlarged up to a #20 file.”

- Page 9, line 135. "the periapical lesions were scanned with a micro-135 CT scanner" the rats were scanned, not the periapical lesions"

Changed to the periapical lesions “of the rats” were scanned with a micro-CT scanner

- What were the parameters used during the scanning procedure?

Scanning procedure performed under the following condition. Field of view: φ10 × 10 mm, voxel size: 20 × 20 × 20 μm, tube voltage: 90 kV, tube current: 160 μA.

We added the information in the materials and methods section.

- What about the radiation in which these rats where submitted during all these scans?
According to the micro-CT (R_mCT2) instruction, one scanning procedure requires 26 sec and radiation exposure level is less than 0.1mGy. This time we had seven opportunities of micro-CT scanning, and each time we needed to scan twice for right and left sides so in total 1.4mGy of radiation exposure became. It is reported that there is a possibility of significant loss of bone density of the lumbar spine three days after 2mGy of radiation exposure, and it will recover after 30 days from the radiation treatment. (Zhai J, He F, Wang J, Chen J, Tong L, Zhu G. Influence of radiation exposure pattern on the bone injury and osteoclastogenesis in a rat model. Int J Mol Med. 2019 Dec;44(6):2265-2275.). Our radiation exposure was lower than this so we think it wouldn’t cause any problem.

Results
- Is there a control group in the micro-ct analysis? The authors did not mention it on the MM section. What is the N of this group?

There is a control group in the micro-CT analysis. The left mandibular first molars of the irradiated group were used as a control group without RCT. We added that information in MM section. N=6 each for micro-CT analysis. Detailed information of rats used in each experiment is shown below.

- This section also needs to be rewritten

We modified this section.

Discussion
- This section is poorly written

We changed the manuscript and English proofreading was performed.

- The authors did not discuss the results of immunohistochemical analysis of the periapical lesion and the quantification of growth factors expression with rat osteoblasts and fibroblasts
We modified the manuscript and added the comments about immunohistological analysis in the discussion.

Figures
- A micro-CT image showing the reduction of the periapical lesion is relevant to present herein

We added representative micro-CT images of each groups in Fig. 2.

Reviewer #3: The use of high frequency waves in endodontic treatment is a hot topic nowadays and this study was well designed. This manuscript focused on the assessment of the functional efficacy of RCT with HFW in vivo with rat model and also in vitro. I appreciate the hard work of the authors to provide important information to the endodontic field regarding HFW.
I have some recommendations and concerns:
1. In introduction line 48, why did authors compared the success rate for initial root canal treatment with pulpectomy? What was the purpose of the comparison of these two procedures?

This part made readers confused so we deleted it.

2. Please check grammar and abbreviations, eg. line 76 micro-computed tomography should be (Micro-CT)

We modified the manuscript as micro-computed tomography (micro-CT).

3. The description of materials and methods needs to be reorganized. The paragraph describing root canal treatment in rats (line 107-131) was unclear and was repeated in the paragraph of quantification of bacteria in the root canal (line 148-173)

We modified the manuscript to make it clear.

4. The paragraph of statistical analysis (line 246-252) needs to be revised. In scientific writing, authors should avoid using "we used...".

We corrected the manuscript following the advice as below.

“Tukey’s test was used to detect statistically significant differences in the volume change of the periapical lesions. The Steel-Dwass test was used for statistics on the quantification of root canal bacteria. Each risk factor was evaluated as 5%. To perform statistical analysis, IBM SPSS Statistics (version 22.0, IBM SPSS, Chicago, IL, USA) was used.”

5. In the discussion, authors discussed the mechanism of HFW's bactericidal effect and its thermal energy generation. Could authors discuss the potential damage of periodontal ligament by using HFW given to the rise of temperature?

We couldn’t observe any damages from rats’ histological sections. Also, we did a preliminary test on the effect of HFW using human periodontal ligament cells and found HFW irradiation never showed damaging effects. Rather, it showed the tendency of cell proliferation. Data of our preliminary experiment is shown below.

Again, I appreciate the authors' contribution and looking forward to your responses.
We really appreciate your comments.

Reviewer #4: The aim of this paper is interesting and the study may provide a new non-surgical treatment for apical periodontitis, however some flaws need to be addressed.
Abstract:
1. Line 38-40, the authors concluded that “high frequency wave irradiation promoted healing of the periapical lesion through increased expression of VEGF, FGF2 and TGF-β1”,however, in the part of ”Immunohistochemical observation” (methods), the samples were stained with specific antibodies against IL-1β, TGF-β1 and FGF2. Please explainhow these results lead to the conclusion described above.

We added the comments below in the discussion to explain how these results lead to the conclusion.

“Yumoto et al. [25] reported that HFW irradiation on mouse osteoblasts induced gene expression of growth factors such as TGF-β1, VEGF, and FGF2 from osteoblasts in vitro. In the present study, the expression of TGF-β1 was up-regulated at the border between the alveolar bone and the lesion in the HFW irradiated group (Fig. 4r, v), as well as FGF2 within the lesion (Fig. 4j, n). This data suggests that HFW promoted the healing of apex lesions by promoting the expression of TGF-β1 and FGF2.

In addition, IL-1β was down-regulated in HFW irradiated group, suggesting that IL-1β suppressed the inflammation in periapical lesions. Further evaluation of the expression of osteoblastic markers and the proliferating cells in the lesions may be needed.”

Introduction:
2. Line 48-49, “It has been reported that the average success rate for initial root canal treatment (RCT) was lower than that of pulpectomy”. This sentence is quite confusing. Please explain this in detail.

This part made readers confused so we deleted it.

Methods:
3. In the parts of “Root canal treatment in rats”, line 128-129, “HFW irradiation was applied three times each to the periapical lesion and the inside of the root canal immediately before the root canal obturation”, however, in the part of “Quantification of bacteria in the root canal”, line 150-151, “HFW irradiation was applied three times to the periapical lesion and the root canal after the root canal preparation”. Are these two time point the same?

Yes, these two time points are indicating exactly the same timing. To avoid confusion, we deleted the description in the part of “Quantification of bacteria in the root canal.”

4. Line 153-154, “Immediately following the RCT, 10 rats were sacrificed and the mandibular first molars were extracted”, the rats were sacrificed and bacteria were collected immediately following the RCT, however, in the corresponding part of result, line 267, “At 4 weeks after RCT”. Authors described the ATP and PCR results for “Quantification of bacteria in the root canal” was from samples from 4 weeks after RCT. Please confirm the descriptions and make the unification.

We confirmed and unified the description about timing.

Results:
5. Line 255-258, “Compared with the controlgroup, the volume of the periapical lesions in the mesial root was significantly smaller in the irradiated group at 2 weeks after the RCT (p = 0.009), and in the non-irradiated group at 6 weeks after the RCT(p = 0.013, Fig. 2)”, authors described that the volume of periapical lesions was significantly smaller in non-irradiated group at 6 weeks after RCT, but form Fig. 2, we could find that at 2 weeks after RCT, the volume of periapical lesions was also significantly smaller in non-irradiated group when compared with the control group (“a” and “b” meant significant difference). Please confirm the result.

We confirmed the result and found the volume of the periapical lesions in the mesial root was significantly smaller in the irradiated group at 2 weeks after the RCT and the letter in Fig. 2 was incorrect so modified the letter in Fig. 2. Thank you very much for your advice.

6. Line 333-335, “In rat osteoblasts, the expression of FGF2 was significantly higher in HFW irradiated osteoblasts than in non-irradiated osteoblasts at 3 days (irradiation applied 5 and 10 times, p = 0.010) and 5 days (irradiation applied 10 times, p = 0.013)”, the authors obtained the result that the expression of FGF2 was significantly higher in HFW irradiated osteoblasts than in non-irradiated osteoblasts at 5 days with 10 times irradiation, however, from Fig. 7(b), we could observed that both for irradiation with 5 times and 10 times at 5 days, expressions of FGF2 showed no significant difference (the same letter “a” meant no significant difference). Please confirm the result.

For better understanding for readers, this time we modified the whole manuscript concentrating on in vivo experimental part to clarify the point so we deleted this part.

7. Line 344-345, “By comparing the expression level of (a) FGF2, (b) VEGF, and (c) TGF-β1 in rat fibroblasts, and (d) FGF2, (e) VEGF, and (f) TGF-β1 in rat osteoblasts”, the authors described that (a), (b) and (c) meant the expression level in rat fibroblasts, and (d), (e) and (f) meant the expression level in rat osteoblasts. However, in Fig. 7, we found t (a), (b) and (c) meant the expression level in rat osteoblasts, and (d), (e) and (f) meant the expression level in rat fibroblasts, which was quite contrary to the description. Please confirm it.

Same as above, we deleted this part to focus in vivo experiments.

Discussion:
8. The irradiation times in all parts of in vivo were consistent (three times each to the periapical lesion and the inside of the root canal), however, in the in vitro assays, the irradiation times were various (15 times for biofilm discs, 5 and 10 times for cells). The authors should discuss why they chose these different irradiation times and whether the different irradiation times affected the therapeutic effect.

Same as above, we modified the manuscript to focus in vivo experiments.

Reviewer #5: The central idea of the manuscript was to access antibacterial/antibiofilm efficacy and growth factors expression to high frequency waves applied during endodontic treatment in teeth with periapical lesions. The manuscript proposal is interesting and focuses on a new possibility for root canal treatment. However, some major changes should be considered.

Abstract
1) Objective is not clear – to “investigate the influence of root canal treatment using high-frequency waves” on…? Antibiofilm effect was not mentioned. Growth factors expression was note mentioned.

We modified throughout the manuscript to clarify the points and focus on in vivo experiment. Then we changed objective as below.

“In the present study, we developed a new high-frequency therapy model in rats based on Yoneda et al. [9], with some modifications, to be used to elucidate the mechanism of action of HFW therapy in apical periodontitis. The influence of HFW as a supplement to conventional root canal therapy on periapical lesions was evaluated by three-dimensional micro-CT analysis, bacteriological evaluation and immunohistochemical analysis.”

2) Methodology should be better explained. It is too superficial. It is not possible to understand how the in vitro e in vivo experiments were performed. It is too confusing.

As this reviewer pointed, our previous manuscript was long and made readers confused with in vitro and in vivo data. This time we focused to investigate the mechanism of High-frequency waves (HFW) therapy which is already reported the efficacy in promoting healing in human clinical studies but no mechanisms are indicated for now by using rat animal model. Following your comment, we also modified the methodology part.

Introduction
1) Introduction should be more concise and objective.

We revised the manuscript.

2) Authors reported: “Recently, it was reported that the balance between bacteria and the host immune system is important for biofilm related infections [13].” Recently, from 2002? It is not recent information.

As this reviewer said this data is not recent so we deleted the word from the manuscript.

3) The reference is missing: “The initial choice for the treatment of apical periodontitis is to remove the source of infection; however, it is speculated that strengthening the host immune system can also lead to healing.”

We added the reference (9-12).

4) Authors reported: “This study focused on high frequency wave (HFW) apical therapy, which is a nonsurgical treatment for apical periodontitis.” Is HFW a nonsurgical treatment for apical periodontitis? Reference?

HFW plays a complementary role to NSRCT procedures. So, we tried to show that HFW is an "adjuvant" to NSRCT. We aimed to investigate the complementary effect of HFW irradiation in addition to NSRCT, which is mechanical / chemical removal of the infectious sources. Also we added the reference (26).

5) Authors reported: “In the dental field, this principle is applied in electric cauterization.” Reference?

Reference are added at that part (16, 17).
6) TGF-β1, VEGF and FGF2. Abbreviation should be described first.
We modified the manuscript to describe abbreviations first.

Materials and Methods
1) Authors reported: “Root canal treatment in rats: Thirty-four 10-week-old male Wistar rats were used for this experiment, and the experimental design is shown in Figure 1.” Information about rats had already been mentioned - unnecessary.

We deleted that expression.

2) Figure 1 should be mentioned before the topic “root canal treatment in rats”.

We modified the manuscript following your advice.

3) Why only the left mandibular first molars of the irradiated group were used as control group without RCT?

To unify the sample number, we chose the left mandibular first molars of the irradiated group as control group.

4) Authors reported: “In both groups, the root canals of the right mandibular first molars were prepared as in conventional RCT.” Which groups?

We performed conventional RCT both in the irradiated group and the non-irradiated group. For irradiated group we added HFW application just before the obturation.

Here we show the detailed information of each rat.

5) What was the tip of the endodontic file used?

We used the K-file, and enlarged to #20. We added the description in the materials and methods section.

6) Both topics “Root canal treatment in rats” and “Quantification of bacteria in the root canal” present information about root canal treatment and HFW.

We deleted the topic in “Quantification of bacteria in the root canal section” to unify.

7) About the topic “Biofilm formation” – “the HA discs…had been treated with saliva for more than 8 h.” Sterile saliva? Normal saliva? More than 8h means 9h, 10h, 24h?? Number of samples?

We deleted this part to focus in vivo experiments and clarify the manuscript.

8) “S. mutans and E. faecalis were cultured for 2 days, and P. intermedia, P. gingivalis and F. nucleatum were cultured for 7 days.” What does it mean?

Same as above, we deleted this part.

9) Authors used different times of HFW irradiation for different experiments (root canal and periapical lesions, biofilm on HA discs, rat cells). Why?

Same as above, we deleted this part.

Results/figure
1) “The effect of HFW radiation on bacterial biofilm in vitro: Observation of the biofilm on a hydroxyapatite (HA) disc with CLSM revealed that P. gingivalis and P. intermedia showed an antibiofilm effect after direct HFW irradiation (Fig. 5a)”. It should be better described. Bacterial species showed antibiofilm effect or HFW irradiation promoted an antibiofilm effect on P. gingivalis and P. intermedia?

Same as above, we deleted this part and concentrated in vivo experiments.

2) Figure
We modified the figures as well for better understanding.

Discussion

1) “This work assessed the influence of HFW irradiation by studying the general frequency, voltage, energization time, and interval of HFW.” It was not the objective of the present study. Authors did not test different frequencies, voltage, energization time and interval…at least it was not mentioned at the methodology. According to HFW treatment device: “Irradiation conditions were set according to the method of Tominaga et al: frequency 510 kHz, voltage 35 V, energization time 1 s, irradiation interval 4 s.” Authors only varied the number of application.

We revised the whole manuscript to clarify. Experimental setting condition information is moved to materials and methods part, and we discussed about that condition in the discussion part.

2) Authors reported: “In previous human studies, as in this rat study, the promotion of healing by HFW after a certain time period following RCT has been consistent [20].” However, reference 20 is a review article related to bone fracture and the conclusion refers to the uncertainty surrounding the use of electrical stimulation and fracture healing.

We changed that reference.

3) Authors did not mention anything about possible side effects or consequences of the HFW.
We couldn’t observe any damages from rats’ histological sections, and for now we haven’t found side effects in our experimental setting. We hypothesized HFW may cause the damage to periodontal ligament, and we conducted a preliminary experiment to see the effect of HFW irradiation on human periodontal ligament cells and found HFW irradiation never showed damaging effect. It rather showed the tendency of cell proliferation. Data of our preliminary experiment is shown below. We added the comment in the discussion part that we couldn’t find any adverse events.

References
1) Most references are out of date.

We changed manuscripts to use the newer references.
