# Supplementary material for: Assessment of the functional efficacy of root canal treatment with high-frequency waves in rats
Source: PLoS One. 2020 Sep 29;15(9):e0239660. doi: 10.1371/journal.pone.0239660 (PMC7523981; doi:10.1371/journal.pone.0239660)
Supplement: Attachment — Submitted filename: Response to Reviewers.docx [file pone.0239660.s003.docx]

Reviewer #2: It was my pleasure to revise again this paper. The authors tried to answer all the questions raised by the reviewers, which improved the quality of this paper. Therefore, I commend the authors on their efforts. However, I still have several concerns/suggestions that need to be adressed

1. Overall, English still needs to be revised

Proofreading by an English native speaker was performed and attached please find the certificate of proofreading.

2. Abstract: this section is confusing. I suggest the authors to start this section with the aim of the manuscript. Then, explain, in a concise way, the methods used.

Following your suggestion, we changed abstract to start with the aim, and then explained the methods used more concisely.

3. Introdution: another section that needs to be revised in order to make it
- Page 9, line 52. Root canal system instead of root canal

We changed the sentence following your advice.

- Page 9, line 54. Remove hand and rotary files as the root canal can be prepared using other kinematics as reciprocation, oscillatory

We removed the phrase “hand and rotary files” following your suggestion.

- Page 9, line 56. Remove the word often

We removed the word “often” following your advice.

- Page 10, line 60. "Removing the source of infection is considered the choice treatment" The authors mentioned it a few line above.

We deleted that part to avoid duplication.

4. Methods:
- Please provide the total amount of irrigation used per tooth (the authors mentioned it in a reply to a reviewer

We revised this part as below.

“Root canals were irrigated with 0.5 ml each time, in total 2ml of 2.5% NaOCl.”

- Why did the authors use RealSeal? This is not fabricated anymore

Real Seal SE (Pentron) was used in this experiment because it was used in the previous study by Yoneda et al. Real Seal SE sealer is currently available for purchase in Japan.

- How was the HFW irradiation applied to the periapical lesion?

We applied once to the periapical lesion and twice to the inside of the root canal as described in materials and methods section as below.

“HFW irradiation was applied once to the periapical lesion and twice to the inside of the root canal immediately before root canal obturation.”

- How was the procedure to assess the same cross-section at the same point in all time-points? Did the authors register the image-stacks?

Image processing software we used this time (TRI/3D-BON) didn’t have the function to register the image-stacks, therefore stacked images were reconstructed by determining a reference point so that they had the same cross section.

5. Discussion:
- Page 20, line 246. This is not correct, as this was not the aim of this paper. Please, rewrite it. Besides, do not use the word biofilm

Following your advice, we revised this part as below.

“This work assessed the influence of HFW irradiation in vivo in a rat model and investigated the target of HFW.”

- Page 22, line 271. "RCT successfully removes most bacteria leaving only that which has invaded deep inside the dentinal tubules". This is incorrect, as several studies already demonstrated that 30-60% of the root canal walls remain untouched after root canal preparation

As this reviewer suggested, it has been reported that residual bacteria in isthmus and lateral branches are difficult to remove by conventional RCT. In a rat root canal treatment model, Yoneda et al. reported that 75% of the bacteria from untreated root canals were eradicated by conventional root canal treatment and led to healing, but the remaining 25% were difficult to remove by conventional RCT only. We modified this part as below.

“Furthermore, residual bacteria decreased significantly after RCT alone, compared with the level before treatment. Yoneda et al. reported that RCT removed 75% of bacteria in the root canal and that 75% of cases were cured with conventional RCT; however, the remaining 25% of bacteria were difficult to remove [26]”

Reviewer #3: Thank you for revising your manuscript and your hardworking is appreciated by the reviewer.
Although the authors addressed most of the previous comments from the reviewer, it is still unclear in the M&M session.

For example, in each paragraph, authors used the same expression of " Twelve rats were divided equally into an irradiated group (n = 6) and a non-irradiated group (n = 6)" twice, and "The rats were divided into an irradiated group (n = 5) and a non-irradiated group (n = 5)." once.
I understand that there were 34 rats in total included in this study, and they were divided into three groups: MicroCT, Quantification of bacteria in the root canal, and Immunohistochemical observation. For each method, there were 12, 10, and 12 subjects included, respectively.
However, the M&M was still not clear and the repetitive description of "Twelve rats were divided equally into an irradiated group (n = 6) and a non-irradiated group (n = 6)" will cause confusion and may require readers to go back and forth to check the subject allocation.
Please revise this session accordingly.

Thank you very much for your comments. Following your suggestion, we revised the description about the number of rats allocated in each experiment. In materials and methods “Animals” section, allocation of all 34 rats are provided and also in each experiment, we modified the description of the number of rats used for better understanding.

Also, the sentence in Line 55 "treatment (RCT) often fails because the anatomical complexity of the root canal system, which includes lateral branches and isthmuses [2] and extraradicular biofilms [3-6], makes it difficult to completely remove the source of the bacterial infection [7]." is still inappropriate. The meaning of this sentence is inexplicit because it can be interpreted as "RCT often fails". In fact, initial NSRCT has a success rate around 95%.

Some have reported a success rate of approximately 95% for NSRCT, but this represents the success rate for pulpectomy in which no lesions have formed at the root apex, and does not represent the success rate of infected root canal treatment of teeth with pulp necrosis and apex lesion formation. The success rate of treatment of pulp-necrotic teeth with preoperative lesions is reported in the following article to be 82%.

Treatment outcome in endodontics: the Toronto study--phase 4　*J endod*,34(3):258-263 2008.

However, we thought it would be better to weaken the tone of the argument, so we modified that part as “Root canal treatment (RCT) can fail due to the anatomical complexity of the root canal system, which includes lateral branches and isthmuses [2], and extraradicular biofilms [3-6], which makes it difficult to completely remove the source of the bacterial infection [7].” to tone down. We appreciate your comments.
